# Modeled Dietary Impact of Pizza Reformulations in US Children and Adolescents

**DOI:** 10.1371/journal.pone.0164197

**Published:** 2016-10-05

**Authors:** Gabriel Masset, Kevin C. Mathias, Antonis Vlassopoulos, Famke Mölenberg, Undine Lehmann, Mike Gibney, Adam Drewnowski

**Affiliations:** 1 Public Health Nutrition, Nestlé Research Center, Lausanne, Switzerland; 2 Department of Human Nutrition, Wageningen University and Research Center, Wageningen, The Netherlands; 3 Institute of Food and Health, University College Dublin, Dublin, Ireland; 4 School of Biomedical Sciences, University of Ulster, Coleraine, United Kingdom; 5 Center for Public Health Nutrition, University of Washington, Seattle, Washington, United States of America; UMR INSERM U866, FRANCE

## Abstract

**Background and Objective:**

Approximately 20% of US children and adolescents consume pizza on any given day; and pizza intake is associated with higher intakes of energy, sodium, and saturated fat. The reformulation of pizza products has yet to be evaluated as a viable option to improve diets of the US youth. This study modeled the effect on nutrient intakes of two potential pizza reformulation strategies based on the standards established by the Nestlé Nutritional Profiling System (NNPS).

**Methods:**

Dietary intakes were retrieved from the first 24hr-recall of the National Health and Nutrition Examination Survey (NHANES) 2011–12, for 2655 participants aged 4–19 years. The composition of pizzas in the NHANES food database (n = 69) were compared against the NNPS standards for energy, total fat, saturated fat, sodium, added sugars, and protein. In a reformulation scenario, the nutrient content of pizzas was adjusted to the NNPS standards if these were not met. In a substitution scenario, pizzas that did not meet the standards were replaced by the closest pizza, based on nutrient content, that met all of the NNPS standards.

**Results:**

Pizzas consistent with all the NNPS standards (29% of all pizzas) were significantly lower in energy, saturated fat and sodium than pizzas that were not. Among pizza consumers, modeled intakes in the reformulation and substitution scenarios were lower in energy (-14 and -45kcal, respectively), saturated fat (-1.2 and -2.7g), and sodium (-143 and -153mg) compared to baseline.

**Conclusions:**

Potential industry wide reformulation of a single food category or intra-category food substitutions may positively impact dietary intakes of US children and adolescents. Further promotion and support of these complimentary strategies may facilitate the adoption and implementation of reformulation standards.

## Introduction

Efforts to improve the diet of US children and adolescents have been focusing on reducing excessive intakes of energy, added sugars, and solid fats; weight management and obesity prevention being top priorities [1–3]. In 2011–12, more than 30% of US children and adolescents were classified overweight or obese; obesity rates were 17.7% for children aged 6 to 11 and 20.5% for adolescents aged 12 to 19 [4].

Increasingly, such efforts are pointing at the potential role of reformulation of processed food products [3, 5–7]. Product reformulation that conform to voluntary or government mandated nutrition standards may complement efforts to change consumer behavior as a cost-effective strategy to improve the overall diet quality [8–11]. For example, following the publication of the 2004 Global Strategy on Diet, Physical Activity and Health [5] and its translation into local policies, many food manufacturers took actions to remove trans fats and lower the sodium content of their products [12, 13]. Nonetheless, better quantifying the potential of more holistic food reformulations − i.e. considering more nutrients − to improve overall nutritional quality of diets is needed to determine if this strategy should be further encouraged [14, 15].

For product reformulation to be maximally effective, it needs to be targeted at the most frequently eaten foods. In the present study, we focused on pizza as it has been consistently ranked among the top energy sources of the US youth in the last two decades [16]. During 2007–10, pizza was consumed by approximately 22% of the 6–19 y age group on any given day [17]. On consumption days, pizza was associated with higher energy, sodium, and saturated fats intake [7]. The feasibility of reformulating pizza products has been assessed, with significant improvements of the nutrient profile of products without altering taste preference [18].

In addition to identifying which foods to reformulate, food manufacturers and the food service industry need guidance in their reformulation efforts, i.e. existing food and nutrient guidelines need to be translated at a product level. Nutrient profiling can be used as tool to guide nutrition-oriented food reformulation [19]. The Nestlé Nutritional Profiling System (NNPS) has been developed specifically for such purpose [20]. It is implemented in all countries in which Nestlé operates (197 in 2015), and was shown to be able to identify more nutritious products across several food categories [21].

This study modeled the potential impact of a pizza reformulation–following the NNPS standards–on nutritional intakes of children and adolescents living in the US.

## Materials and Methods

### Study population

The population for the present study was taken from the 2011–12 NHANES survey, an ongoing program designed to assess the health and nutritional status of the US population by using a multi-stage sampling design. Dietary intakes were assessed through 24-hour recalls, sociodemographic data as well as clinical measures were also collected, as described elsewhere [22]. NHANES 2011–12 was approved by the National Center for Health Statistics Research Ethics Review Board; every participant and/or parent or guardian provided written informed consent.

For the present study and in accordance with the Nestlé Nutritional Profiling System (NNPS–see below), we restricted the sample to all individuals aged between 4 and 19, not breastfeeding, and having completed the first 24-hour recall. This 24-hour recall was conducted by a trained dietary interviewer in mobile examination centers. Participants aged 12 years and older completed their own dietary interviews, children aged 6 to 11 completed proxy-assisted interviews, and children below 6 had their intake reported by a proxy respondent.

### Identification of pizza food codes and effect of pizza consumption at baseline

For each individual, energy (kcal), total fat (g), saturated fat (g), sodium (mg), total sugars (g), and added sugars (g) intakes were calculated by combining the declared quantity (grams) of food intake with the 2011–12 Food and Nutrition Database for Dietary Studies (FNDDS) composition table [23]. Added sugars intakes were obtained through the complementary Food Patterns Equivalents Database files [24].

Based on description, we identified 69 pizzas in the FNDDS (S1 Table). Prevalence of pizza consumption was defined as reporting intake of a pizza food code on any eating occasion during the first 24-hour recall. We examined prevalence of consumption by age group (children aged 4 to 11 years and adolescents aged 12 to 19 years), gender, ethnicity, and education level of the head of household.

### The Nestlé Nutritional Profiling System (NNPS) and its application to the FNDDS

The NNPS was specifically designed to guide the reformulation of Nestlé food and beverage portfolio, as described in details elsewhere [20]. Briefly, the NNPS is a category-specific system that calculates nutrient targets per serving as consumed, based on age-adjusted dietary guidelines. Excessive amounts of nutrients to limit cannot be compensated for by adding nutrients to encourage. Nutrients to limit are similar across the food categories (eg, total and saturated fats, sodium, added sugars); while nutrients to encourage are category-specific (eg, protein in the case of pizzas).

In this study, we used nutrient targets defined for the ‘Pizza as a center of plate’ category (targets displayed in the results section) and applied these to all FNDDS pizza codes, using the RACC portion size of 140g [25]. Mean nutrient content of pizzas consistent with the NNPS standards were compared with those not meeting the standards using unpaired t-tests.

### Reformulation and substitution scenarios

Two scenarios were created to simulate the impact of a potential pizza reformulation following the NNPS standards.

First, a reformulation scenario applied the specific NNPS nutrient targets on the current FNDDS pizza, thereby simulating the minimum reformulation needed to achieve the NNPS standards. For example, if ‘pizza x’ energy content was above 286 kcal/100g (the NNPS target), it was set to such value. NNPS targets were applied independently, i.e. when modifying one nutrient value, there was no adjustment made on other values (e.g. if saturated fat was reduced, energy may have been left unchanged if meeting the respective NNPS target). Since such scenario may not reflect how pizza products would be effectively reformulated–reformulated pizza products being purely theoretical–a substitution scenario simulated a shift to existing pizzas that met NNPS standards.

In the substitution modeling, pizzas not meeting the NNPS standards were replaced with a pizza meeting the standards. The substitution of a pizza not meeting the NNPS standards was made on a one-to-one basis, by selecting the closest pizza from a nutrient standpoint meeting the NNPS standards. The closest pizza was identified by minimizing the sum of the differences for all nutrients of interest based on the nutrient content per 100g (pdist function in R):

$${\text{Distance}(\text{Pizza}}_{1}{,\;\text{Pizza}}_{2})=\sqrt{\sum_{\text{NNPS factors}}^{}{{(\text{Content Pizza}}_{1}-{\text{Content Pizza}}_{2})}^{2}}$$

One-to-one substitution couples are given in the Supporting Information (S2 Table).

### Intakes at baseline and in modeled diets

Mean daily intakes of energy, total and saturated fat, sodium, protein, and total and added sugars of pizza consumers at baseline (i.e. observed intakes) were compared with intakes in the reformulation scenario, the substitution scenario, and among pizza non-consumers. Further, we analyzed the proportion of participants being over the maximum recommended intakes of saturated fat and sodium, using recommendations from the Dietary Guidelines for Americans 2015 and the Institute of Medicine, respectively [3, 26].

To take into consideration the sampling design of NHANES, all analyses were conducted using the survey package of the R software (version 3.2.1).

## Results

### Prevalence of pizza consumption

Within the selected sample (n = 2655), 20.9% of children and adolescents reported eating pizza (Table 1). There was no significant difference in terms of consumption prevalence between age groups, genders, education of the head of household, or ethnicity. Results were similar within children and adolescents (S3 Table).

**Table 1:** Prevalence of pizza consumption in NHANES 2011–12 children and adolescents having completed the first 24-hr recall (Day 1), by gender, age group, education level, and ethnicity.

	n	% consuming pizza^a^	p
**Total**	2655	20.9	
**Gender**			0.541
**Females**	1298	18.9	
**Males**	1357	21.4	
**Age group**			0.865
**Children (4-11y)**	1503	20.6	
**Adolescents (12-19y)**	1152	21.1	
**Household education**			0.513
**Up to high school**^**b**^	2000	20.0	
**College and above**^**b**^	579	26.6	
**Ethnicity**^**c**^			0.743
**White**	605	21.9	
**Mexican**	504	21.2	
**Black**	784	18.8	
**Asian**	317	18.5	

^a^ Pizza consumption was defined as having declared consuming a pizza food code at least once during Day 1. % and chi-square tests were adjusted based on sampling design, using the ‘survey’ package of R

^b^ Self-reported education level of the head of the household

^c^ Ethnicity was self-reported

The average reported intake of pizza was 209 g among pizza consumers, and 42 g in the whole study sample. Pizza contributed to 27% of total calories among pizza consumers, and to 6% of total calories in our study sample; pizza contribution to saturated fat and sodium intake was higher (Table 2).

**Table 2:** Mean daily intake from pizza products in NHANES 2011–12 children and adolescents aged 4 to 19 years (Day 1).

	Intake from pizza					
	Total population (n = 2655)			Pizza consumers^a^ (n = 579)		
Nutrient	Mean	SE	% daily intake	Mean	SE	% daily intake
**Energy (kcal)**	122	12.8	5.95	584	42.8	27.0
**Total fat (g)**	5.34	0.56	7.03	25.6	1.99	32.4
**Saturated fat (g)**	2.18	0.23	8.35	10.5	0.80	36.6
**Sodium (mg)**	268	27.9	8.19	1285	94.9	36.3
**Protein (g)**	5.29	0.55	7.27	25.4	1.88	33.4
**Total sugars (g)**	1.53	0.15	1.17	7.36	0.47	5.57
**Added sugars (g)**	0.42	0.05	0.50	2.03	0.19	2.42

^a^ Pizza consumption was defined as having declared consuming a pizza food code at least once during Day 1

### Classification of pizzas according to the NNPS

Of the 69 pizza codes in the FNDDS, 20 were consistent with the NNPS standards (29%, Table 3), i.e. they met all NNPS nutrient targets. The most limiting NNPS nutrient targets, i.e. those that were reached less often, were sodium and total fat; the added sugars target was the least limiting. Pizzas that were consistent with the NNPS standards contained on average less total and saturated fat, sodium, energy, protein, calcium, zinc, vitamin A, vitamin B12 and vitamin E compared to Fail-pizzas (Table 3 and S4 Table); but had higher nutrient density per kcal for fibers, potassium, magnesium, and vitamin C (S5 Table). For most types of pizza flavors (as described in the FNDDS), there were products consistent and not consistent with the NNPS standards (S1 Table). Among pizza consumers, 19% reported consuming only pizzas consistent with NNPS standards.

**Table 3:** Mean nutrient content per 100 g of pizza food codes in the FNDDS 2011–12 database, by NNPS standards ^a^.

	All pizzas (n = 69)		Pizzas not meeting NNPS standards(n = 49)		Pizzas meeting NNPS standards (n = 20)				
Nutrient	Mean	SE	Mean	SE	Mean	SE	p_Fail-Pass_	NNPS Nutrient target (per 100g)	% of pizzas achieving specific NNPS nutrient target
**Energy (kcal)**	268	4.03	280	4.66	241	3.31	< .001	≤286	82.6
**Total fat (g)**	11.6	0.35	12.7	0.40	9.09	0.20	< .001	≤40% energy ^b^ or ≤7.5	59.4
**Saturated fat (g)**	4.78	0.16	5.21	0.19	3.72	0.09	< .001	≤17.5% energy ^b^or ≤2.5	73.9
**Sodium (mg)**	586	10.7	614	13.0	517	5.69	< .001	≤566 mg	42.0
**Protein (g)**	11.7	0.22	12.1	0.27	10.7	0.27	0.001	≥12% energy ^b^or ≥3.6	97.1
**Total sugars**^**c**^ **(g)**	3.75	0.14	3.55	0.16	4.24	0.29	0.046	N/A	N/A
**Added sugars (g)**	1.06	0.14	0.97	0.14	1.29	0.33	0.381	≤10% energy ^b^or ≤3.6	100

^a^ NNPS: Nestlé Nutritional Profiling System. The NNPS defines category-specific nutrient targets per portion size. All targets need to be met to be consistent with the NNPS standards.

^b^ % energy: Nutritional factor’s percentage of energy contribution in the product

^c^ Total sugars were not defined as targets for the NNPS pizza category, and are provided for information.

### Observed and modeled nutrient intakes

Among pizza consumers, the reformulation scenario resulted in decreases in total energy (14kcal or 0.6%), total fat (1.2g or 1.6%) and saturated fat (0.3g or 1.1%) as compared to baseline (Table 4). The reduction of sodium intake was higher (143mg or 4%), highlighting the effect of this more limiting NNPS nutrient target. The substitution modeling led to larger reduction, with intakes of energy, total and saturated fat, and sodium significantly lowered by 45 kcal (2%), 6.8 g (8.6%), 2.7 g (9.5%), and 153 mg (4.4%), respectively.

**Table 4:** Mean nutritional intakes in NHANES 2011–12 children and adolescents aged 4 to 19 years (Day 1), at baseline and in the reformulation and substitution scenarios.

	Pizza consumers (n = 579) ^a^						Non pizza consumers at baseline (NON, n = 2076)				
	Baseline (BASE)		Reformulation scenario (REF) ^b^		Substitution scenario (SUB) ^c^				p_NON vs BASE_	p_NON vs REF_	p_NON vs SUB_
Nutrient	Mean	SE	Mean	SE	Mean	SE	Mean	SE			
**Energy (kcal)**	2167	75.8	2153	75.5*	2122	73.1*	2018	29	0.099	0.128	0.224
**Total fat (g)**	79.2	4.15	78.0	4.08*	72.4	3.69*	75.1	1.62	0.369	0.500	0.492
**Total fat (% energy)**	32.1	0.88	31.8	0.87*	30.0	0.84*	32.8	0.23	0.432	0.304	0.003
**Saturated fat (g)**	28.6	1.44	28.3	1.43*	25.9	1.28*	25.5	0.65	0.103	0.132	0.800
**Saturated fat (% energy)**	11.6	0.29	11.6	0.29*	10.8	0.28*	11.2	0.16	0.249	0.292	0.270
**Sodium (mg)**	3541	169	3398	158*	3388	158*	3203	56.7	0.084	0.279	0.302
**Protein (g)**	75.8	3.35	75.9	3.35*	76.3	3.48*	72	1.14	0.309	0.313	0.266
**Protein (% energy)**	14.0	0.29	14.1	0.29*	14.3	0.31*	14.4	0.16	0.163	0.253	0.955
**Total sugars (g)**	132	3.85	132	3.85*	136	3.78*	131	2.6	0.766	0.777	0.313
**Total sugars (% energy)**	25.4	1.40	25.6	1.41*	26.5	1.37*	26.3	0.31	0.493	0.558	0.869
**Added sugars (g)**	83.8	3.91	83.7	3.90*	84.2	3.77*	84.4	2.05	0.875	0.869	0.950
**Added sugars (% energy)**	15.8	1.05	15.8	1.04*	16.1	1.02*	16.7	0.28	0.413	0.452	0.602

^a^ Pizza consumption was defined as having declared consuming a pizza food code at least once during Day 1.

^b^ In the Reformulation scenario, if the nutrient content of a pizza was not consistent with NNPS target, it was set to the NNPS target for this nutrient.

^c^ In the Substitution scenario, all pizzas not consistent with NNPS standards were replaced by the closest pizza consistent with NNPS, based on a Euclidean distance calculated using all NNPS nutritional factors.

* Due to the modeling, all differences in nutrient intakes between baseline and the reformulation and substitution scenarios were highly significant (all p_BASE-REF_ and p_BASE-SUB_ < .001).

NNPS, Nestlé Nutritional Profiling System. The NNPS defines category-specific nutrient targets per portion size. All targets need to be met to be consistent with the NNPS standards.

In both the reformulation and substitution scenarios, trends were similar when intakes of fats, protein, and sugars were expressed in percent of energy, with saturated fat intake going from 11.6 to 10.8% energy in the substitution scenario (Table 4). In addition, trends were similar by age group and gender (S6 Table). In the total sample (pizza consumers and non-consumers), the two reformulation scenarios showed trends in similar directions but with smaller effect size (S7 Table). All reported reductions were highly significant (p<0.001) due to the forced modeling.

At baseline, pizza consumers had higher daily energy (139kcal), sodium (338mg), and saturated fat intake (3.1g) compared to non-consumers (Table 4). These differences were attenuated by up to 30% for energy, 45% for sodium, and 87% for saturated fat in the substitution scenario. Expressed in percent of energy, pizza consumers in the substitution scenario had lower intake of saturated fat than non-consumers.

**Fig 1:**
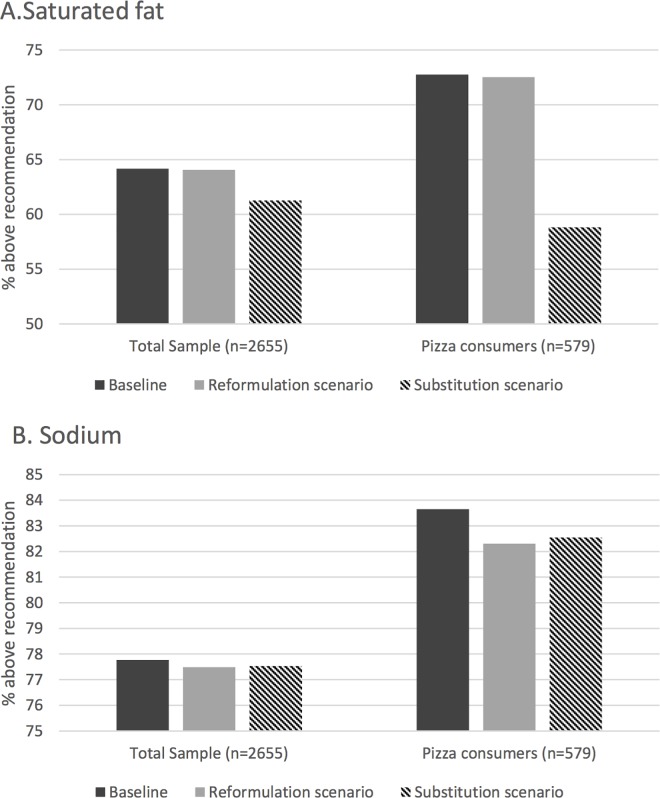
**Proportion of children and adolescents above the recommendations for saturated fat (A) and sodium (B) recommendation, at baseline and in the reformulation and substitution scenarios.** In the Reformulation scenario, if the nutrient content of a pizza was not consistent with NNPS target, it was set to the NNPS target for this nutrient. In the substitution scenario, all pizzas not consistent with NNPS standards were replaced by the closest pizza consistent with NNPS, based on a Euclidean distance calculated using all NNPS nutritional factors. NNPS, Nestlé Nutritional Profiling System. The NNPS defines category-specific nutrient targets per portion size. All targets need to be met to be consistent with the NNPS standards.

In line with Table 4, the modeled reduction of saturated fat and sodium intake in the reformulation and substitution scenarios led to a lower proportion of the population being above recommended intakes compared to baseline (Fig 1). In particular, the proportion of pizza consumers being above the saturated fat recommendation (<10% energy) was reduced between baseline and the substitution scenario (73 versus 59%).

## Discussion

Using the latest NHANES survey with available dietary data, we showed that pizza consumption was high among US children and adolescents, with an intake prevalence of 20.9% in our sample. Such prevalence was lower than in the previous NHANES surveys, confirming the downward trends in pizza consumption previously observed [7, 16]. In our study sample and consistent with previous observations, pizza consumers had higher intakes of total energy, saturated fat, and sodium [7, 17]. At baseline, 64% of children and adolescents were above the daily recommended intake of saturated fat, and 78% were above the sodium recommendation, highlighting that the US youth diet remains unbalanced despite improvements recently observed in the general population [27].

We based our reformulation and substitution scenarios on the Nestlé Nutrient Profiling System (NNPS) [20], for which 29% of FNDDS pizzas passed the standards, showing that the NNPS standards set for the pizza category are achievable but challenging, and would therefore represent realistic and relevant reformulation targets. Fail-pizzas had a higher energy, sodium, and saturated fat content per 100g, as well as higher content per 100 g of protein, calcium, and other micronutrients–i.e. mainly dairy or meat sourced nutrients. While Fail-pizzas provided more nutrients per 100g they also provided more energy per 100g and were thereby less nutrient dense than Pass-pizzas. These results indicate that pizza reformulation efforts and intra category substitutions may not be straight forward from a holistic standpoint. For example, removing an extra cheese topping or choosing a pizza with less cheese could reduce energy, but also reduce calcium intake. Improving the healthfulness of pizzas based on energy, sodium, saturated fat, or added sugar content could result in pizzas with lower contents of shortfall nutrients from one ingredient while it might promote others, e.g. fibers from vegetables. Given that pizza is a major contributor to the US youth diets, these specific findings should not be used to discourage future reformulations, but rather to encourage strategies that maximize energy density reduction and increase shortfall nutrients when reformulating pizzas. As previously highlighted by Combet and colleagues, the overall nutrient profile of pizza products can be improved beyond macronutrients [18].

The two scenarios aimed to assess the impact of potential industry-wide pizza reformulations following the NNPS standards. The reformulation scenario was the most conservative. It tested the effect of the NNPS standards per se, assuming that any pizza reformulation would be done to reach exactly the specific nutrient targets; in fact, the reformulated products in the reformulation scenario are purely theoretical. As a result, the modeled dietary impact of the reformulation scenario was in line with the NNPS standards: sodium and saturated fat were the most limiting nutrient targets and higher intake reduction of were observed for these two nutrients. However, effective reformulation to meet the NNPS standards is unlikely to result in products reaching exactly every single nutrient target. For example, multiple ingredients may need to be replaced which will affect all nutrients. The substitution scenario, by selecting existing pizzas consistent with the NNPS standards, overcomes such potential limitation. This substitution scenario led to higher intake reduction of all assessed nutrients compared to the reformulation scenario, suggesting indeed that to reach the NNPS standards, food developers need to reduce nutrient composition of products below the specific NNPS targets. Yet, the nutritional distance calculation used to identify the substitution products may not reflect taste and sensory attributes, and the actual changes for the consumers would need to be further assessed.

Henceforth, a real impact of a pizza reformulation following the NNPS standards should lie in between the reduction estimates we obtained in this study. In both the reformulation and substitution scenarios, pizza consumers had lower intakes of energy (-14 and -45kcal, respectively), saturated fat (-1.2 and -2.7g, respectively), and sodium (-143 and -153mg, respectively), compared to baseline intakes. The effect of pizza substitution was particularly strong for saturated fat, with the proportion of children and adolescents exceeding recommended intakes passing from 73% to 59% among pizza consumers. With regards to energy, the estimated 45kcal reduction reached in the substitution modeling was close to the amount suggested to avert the increase in childhood obesity [28] and was half the recently estimated energy deficit needed to reduce childhood overweight and obesity in Australia [29]. Nonetheless, such reductions would need to be sustained on a daily basis to have relevant public health impact. Our analysis focused on a single food category, and showed that the potential effect of pizza reformulation alone was limited at a population level (S6 Table). Modeling similar reformulation and substitutions on a wider range of food products could further inform on the potential of food reformulation to complement other measures needed to improve dietary balance of US children and adolescents. In particular, portion size selection could play a crucial role in achieving healthier diets and reducing obesity prevalence [30, 31].

The results of the present study confirmed that reformulation of a single food could have some impact on the nutritional intake of children and adolescents [8, 9]. Such effect could be further associated with improved health status of the general population, in particular if reformulation is applied to key nutrients in highly consumed food categories [32, 33]. Since consumption patterns are not global, food manufacturers and regulators may need to focus their efforts or policies towards specific food categories and nutrients that would address best local needs. To identify which reformulation strategies could have the highest public health impact, further research needs to assess the potential effect of reformulating pizza products and other food categories among other populations and regions.

The present study assessed the effect of potential reformulation based only on the NNPS standards. While the NNPS was specifically designed to guide reformulation [20], several other nutrient profiling systems were also suggested for setting reformulation targets [8, 9]. Applying a nutrient profiling system with lower reformulation targets for nutrients to limit could have led to higher reductions of energy sodium and saturated fat in the modeled diets. One limitation of the NNPS, as mentioned above, is that it does not provide criteria for many micronutrients; applying a system taking into account more micronutrients would ensure an overall improvement of the nutritional profile when reformulating products. Nevertheless, the conclusions we reached are strengthened by the fact that the NNPS is currently applied across a very broad food and beverage portfolio, with reformulation targets defined for a wide range of food categories. As an example, almost a third of FNDDS pizzas did reach the NNPS standards, and results were similar across three other categories [21]. The potential nutritional effects estimated in the present study may therefore be realistic providing the whole food supply would adhere to the standards defined by the NNPS.

While such scenario remains possible, it would need time to become effective. First, only 19% of pizza consumers in our sample reported consuming a pizza meeting NNPS standards, i.e. consumers were less likely to opt for pass pizzas which represented 29% of the NHANES food database. This low consumption could be explained by a lower taste preference, a lower availability (e.g. only in schools or some bigger supermarkets), or a higher price for these more nutritious options. Further research is therefore needed to analyze the physical and economic availability of more nutritious options within specific categories and to better understand the consumers’ preferences, to ensure that reformulated products remain available and consumed, or even preferred. Pizza products appear to be particularly suited for reformulation purposes, with nutritional improvements achievable while keeping a high consumer acceptability of the new products [18, 34]. Work conducted by sensory science researchers [34, 35] would need to be included in the public health nutrition agenda and complemented by developing technological means to allow the required reformulations. Second, NNPS nutrient targets are challenging: in Nestlé, 56% of the US pizza portfolio achieved the targets as of July 2015, despite 4 years of NNPS guided reformulation efforts. A stronger and multi-stakeholder framework, as put in place for several front-of-pack logo schemes [36] and proposed at the European Union level [37], is needed to push all manufacturers to reformulate their portfolios while considering technological and cost constraints [38].

Our analyses had some further limitations. In particular, the FNDDS food composition database may not reflect accurately foods currently consumed in the US. For some nutrients, the use of nutrition facts data could help in making sure the latest nutritional values are used when analyzing reformulation potential against the current situation. The inclusion of added sugars in the NNPS algorithm made it necessary to use the FPED files attached to the FNDDS database, the calculations and hypothesis used to derive the added sugar content of all foods in the FNDDS may also present some error in some specific categories. While such limitation was not of primary concern in pizza product, it may affect conclusions obtained when analyzing other food categories. Last, considering the various interview methods for children, the use of 24-hr recall data may have led to reporting bias. Thereby, the estimates we obtained should be interpreted cautiously.

## Conclusion

This modeling study confirmed that reformulation of pizza consumed by US children and adolescents could improve their nutritional intake, relying on an established and implemented nutrient profiling system–the NNPS–and on the selection of pizza items already achieving the standards defined by the NNPS. Food reformulation could play a key role in rebalancing dietary intakes if the present results were confirmed when including reformulation efforts in other food categories and in other countries. In addition, and to ensure that reformulated products do provide an overall improved nutritional profile, reformulation standards would need to consider energy content and both macro- and micronutrients. Further research needs also to account for consumer behaviors to understand how and why individuals would opt for products with improved nutrient profiles and how to guide them towards these options. Cultural backgrounds play a key role in food selection, and there is strong need to assess which population could benefit most from food reformulation, and which food categories should be prioritized.

## Supporting Information

S1 Table Pizzas description and classification according to the NNPS (1 = Meeting standards; 0 = Not meeting standards). (DOCX)

S2 Table Pizza Substitution Table. (DOCX)

S3 Table Prevalence of pizza consumption in NHANES 2011–12 children and adolescents having completed the first 24-hr recall (Day 1), by age group, gender, education level, and ethnicity. (DOCX)

S4 Table Mean nutrient content per 100 g of pizza food codes in the FNDDS 2011–12 database, by NNPS standards. (DOCX)

S5 Table Mean nutrient content per 100 kcal or as % energy of pizza food codes in the FNDDS 2011–12 database, by NNPS standards. (DOCX)

S6 Table Mean nutritional intakes in NHANES 2011–12 children and adolescents aged 4 to 19 years consuming pizza (Day 1), at baseline and in the reformulation and substitution scenarios. (DOCX)

S7 Table Mean nutritional intakes expressed in percent of energy in NHANES 2011–12 children and adolescents aged 4 to 19 years (Day 1), at baseline and in the reformulation and substitution scenarios. (DOCX)

## References

[1] Krebs-Smith SM, Guenther PM, Subar AF, Kirkpatrick SI, Dodd KW. Americans do not meet federal dietary recommendations. J Nutr. 2010;140(10):1832–8. 10.3945/jn.110.124826 20702750 PMC2937576

[2] U.S. Department of Agriculture, U.S. Department of Health and Human Services. Dietary Guidelines for Americans, 2010. 7th Edition Washington, DC: U.S. Government Printing Office, 2010. 10.3945/an.111.000430 PMC3090168 22332062

[3] U.S. Department of Health and Human Services, U.S. Department of Agriculture. 2015–2020 Dietary Guidelines for Americans. 8th Edition. 2015 [Accessed 15 January 2016]. Available: http://health.gov/dietaryguidelines/2015/guidelines/.

[4] Ogden CL, Carroll MD, Kit BK, Flegal KM. Prevalence of childhood and adult obesity in the United States, 2011–2012. JAMA. 2014;311(8):806–14. 10.1001/jama.2014.732 24570244 PMC4770258

[5] World Health Organization. Global Strategy on Diet, Physical Activity and Health. Geneva: World Health Organization, 2004.

[6] World Health Organization. WHO European action plan for food and nutrition policy 2007–2012 Geneva: WHO Regional Office for Europe, 2008.

[7] Powell LM, Nguyen BT, Dietz WH. Energy and nutrient intake from pizza in the United States. Pediatrics. 2015;135(2):322–30. 10.1542/peds.2014-1844 25601973 PMC4306796

[8] Roodenburg AJ, van Ballegooijen AJ, Dotsch-Klerk M, van der Voet H, Seidell JC. Modelling of Usual Nutrient Intakes: Potential Impact of the Choices Programme on Nutrient Intakes in Young Dutch Adults. PLoS One. 2013;8(8):e72378 10.1371/journal.pone.0072378 24015237 PMC3756057

[9] Trichterborn J, Drossard C, Kersting M, Harzer G, Kunz C. The potential impact of nutrient profiles on dairy-related energy and nutrient intake in German children and adolescents. Eur J Clin Nutr. 2012;66(4):466–73. 10.1038/ejcn.2011.180 22045224

[10] Gortmaker SL, Wang YC, Long MW, Giles CM, Ward ZJ, Barrett JL, et al Three Interventions That Reduce Childhood Obesity Are Projected To Save More Than They Cost To Implement. Health Aff. 2015;34(11):1932–9. 10.1377/hlthaff.2015.0631 26526252 PMC9627551

[11] Leroy P, Requillart V, Soler LG, Enderli G. An assessment of the potential health impacts of food reformulation. Eur J Clin Nutr. 2015 10.1038/ejcn.2015.201 26669572

[12] Kloss L, Meyer JD, Graeve L, Vetter W. Sodium intake and its reduction by food reformulation in the European Union—A review. NFS Journal. 2015;1:9–19. 10.1016/j.nfs.2015.03.001

[13] Downs SM, Thow AM, Leeder SR. The effectiveness of policies for reducing dietary trans fat: a systematic review of the evidence. Bull World Health Organ. 2013;91(4):262–9H. 10.2471/BLT.12.111468 23599549 PMC3629452

[14] Combris P, Goglia R, Henini M, Soler LG, Spiteri M. Improvement of the nutritional quality of foods as a public health tool. Public Health. 2011;125(10):717–24. 10.1016/j.puhe.2011.07.004 21890152

[15] Réquillart V, Soler LG. Is the reduction of chronic diseases related to food consumption in the hands of the food industry? European Review of Agricultural Economics. 2014;41(3):375–403. 10.1093/erae/jbu010

[16] Slining MM, Mathias KC, Popkin BM. Trends in food and beverage sources among US children and adolescents: 1989–2010. J Acad Nutr Diet. 2013;113(12):1683–94. 10.1016/j.jand.2013.06.001 23916972 PMC3905608

[17] Rhodes DG, Adler ME, Clemens JC, LaComb RP, Moshfegh Aj. Consumption of Pizza—What We Eat in America, NHANES 2007–10. 2014 Contract No.: 11. 36913513

[18] Combet E, Jarlot A, Aidoo KE, Lean ME. Development of a nutritionally balanced pizza as a functional meal designed to meet published dietary guidelines. Public Health Nutr. 2014;17(11):2577–86. 10.1017/S1368980013002814 24160252 PMC10282456

[19] McColl K, Lobstein T. Nutrient Profiling: Changing the food of Britain London: Coronary Prevention Group and World Obesity Federation, 2015.

[20] Vlassopoulos A, Masset G, Rheiner Charles V, Hoover C, Chesneau-Guillemont C, Leroy F, et al A nutrient profiling system for the (re)formulation of a global food and beverage portfolio Eur J Nutr. 2016 10.1007/s00394-016-1161-9 26879847 PMC5346408

[21] Masset G, Vlassopoulos A, Potter M, Leroy F, Vecchio BM. Improving The US Food Environment Through Reformulation: A Comparison Of Two Nutrient Profiling Models. The FASEB Journal. 2015;29(S1):903.14.

[22] Centers for Disease Control and Prevention (CDC). National Health and Nutrition Examination Survey 2015 [Accessed 25 November 2015]. Available: http://www.cdc.gov/nchs/nhanes.htm.

[23] Food Survey Research Group. Food and Nutrient Database for Dietary Studies 2011–2012: USDA Agricultural Research Service; 2014 [Accessed 17 February 2016]. Available: http://www.ars.usda.gov/SP2UserFiles/Place/80400530/pdf/fndds/fndds_2011_2012.pdf.

[24] USDA Agricultural Research Service. Food Patterns Equivalents Database—Databases and SAS Data Sets 2014 [Accessed 25 November 2015]. Available: http://www.ars.usda.gov/Services/docs.htm?docid=23869.

[25] Sec. 101.12 Reference amounts customarily consumed per eating occasion - 21CFR101.12, (2015).

[26] Insitute of Medicine. Table: DRI Values Summary 2015 [Accessed 30 November 2015]. Available: http://iom.nationalacademies.org/~/media/Files/Activity%20Files/Nutrition/DRIs/5_Summary%20Table%20Tables%201-4.pdf.

[27] Wang DD, Li Y, Chiuve SE, Hu FB, Willett WC. Improvements In US Diet Helped Reduce Disease Burden And Lower Premature Deaths, 1999–2012; Overall Diet Remains Poor. Health Aff. 2015;34(11):1916–22. 10.1377/hlthaff.2015.0640 26526250 PMC4783149

[28] Wang YC, Orleans CT, Gortmaker SL. Reaching the healthy people goals for reducing childhood obesity: closing the energy gap. Am J Prev Med. 2012;42(5):437–44. 10.1016/j.amepre.2012.01.018 22516482

[29] Cochrane T, Davey R, de Castella FR. Estimates of the energy deficit required to reverse the trend in childhood obesity in Australian schoolchildren. Aust N Z J Public Health. 2015 10.1111/1753-6405.12474 26561382 PMC5072353

[30] Marteau TM, Hollands GJ, Shemilt I, Jebb SA. Downsizing: policy options to reduce portion sizes to help tackle obesity. BMJ. 2015;351:h5863 10.1136/bmj.h5863 26630968

[31] Dobbs R, Sawers C, Thompson F, McKenna S, Nuttal R, Spatharou A. Overcoming obesity: An initial economic analysis McKinsey Global Institute, 2014.

[32] Bruins MJ, Dotsch-Klerk M, Matthee J, Kearney M, van Elk K, Weber P, et al A Modelling Approach to Estimate the Impact of Sodium Reduction in Soups on Cardiovascular Health in the Netherlands. Nutrients. 2015;7(9):8010–9. 10.3390/nu7095375 26393647 PMC4586570

[33] Ma Y, He FJ, Yin Y, Hashem KM, MacGregor GA. Gradual reduction of sugar in soft drinks without substitution as a strategy to reduce overweight, obesity, and type 2 diabetes: a modelling study. Lancet Diabetes Endocrinol. 2016 10.1016/S2213-8587(15)00477-5 26777597

[34] Manickavasagan A, Reicks M, Singh V, Sawsana A, Intisar AM, Lakshmy R. Acceptability of a reformulated grain-based food: Implications for increasing whole grain consumption. Food Science and Human Wellness. 2013;2(3–4):105–12. 10.1016/j.fshw.2013.06.002

[35] Tritt A, Reicks M, Marquart L. Reformulation of pizza crust in restaurants may increase whole-grain intake among children. Public Health Nutr. 2015;18(8):1407–11. 10.1017/S1368980014001724 25157427 PMC10271276

[36] Choices Programme. Positive logos present themselves together 2016 [Accessed 26 July 2016]. Available: https://www.youtube.com/watch?v=b7SvKs9GI10,http://choicesprogramme.org/news-updates/news/positive-logos-present-themselves-together.

[37] Dutch Presidency EU Conference for Food Product Improvement. Roadmap for Action on Food Product Improvement: Dutch Ministry of Health, Welfare and Sport; 2016 [Accessed 26 July 2016]. Available: https://www.rijksoverheid.nl/binaries/rijksoverheid/documenten/formulieren/2016/02/22/roadmap-for-action-on-food-product-improvement/roadmap.pdf.

[38] Buttriss JL. Food reformulation: the challenges to the food industry. Proc Nutr Soc. 2013;72(1):61–9. 10.1017/S0029665112002868 23228239

