# Supplementary material for: Modeled Dietary Impact of Pizza Reformulations in US Children and Adolescents
Source: PLoS One. 2016 Oct 5;11(10):e0164197. doi: 10.1371/journal.pone.0164197 (PMC5051708; doi:10.1371/journal.pone.0164197)
Supplement: S1 Table — (DOCX) [file pone.0164197.s001.docx]

**Supporting Information - S1 Table**

| **Pizzas description and classification according to the NNPS (1=Meeting standards; 0=Not meeting standards)** | | | | |  |  |  |  |
| --- | --- | --- | --- | --- | --- | --- | --- | --- |
| NNPS, Nestlé Nutritional Profiling System. The NNPS defines category-specific nutrient targets per portion size. All targets need to be met to be consistent with the NNPS standards. | | | | | | | |  |
| **Source: FNDDS 2011-12** |  |  |  |  |  |  |  |  |
|  | **Overall** |  | **Specific nutrient targets** | | |  |  |  |
| **Pizza name** | **NNPS Outcome** |  | **Energy (kcal)** | **Total fat** | **Saturated fat** | **Sodium** | **Added sugars** | **Protein** |
| Pizza with beans and vegetables, thick crust | 1 |  | 1 | 1 | 1 | 1 | 1 | 1 |
| Pizza with beans and vegetables, thin crust | 1 |  | 1 | 1 | 1 | 1 | 1 | 1 |
| Pizza with cheese and extra vegetables, regular crust | 1 |  | 1 | 1 | 1 | 1 | 1 | 1 |
| Pizza with cheese and extra vegetables, thick crust | 1 |  | 1 | 1 | 1 | 1 | 1 | 1 |
| Pizza with cheese and extra vegetables, thin crust | 0 |  | 1 | 0 | 0 | 0 | 1 | 1 |
| Pizza with extra meat and extra vegetables, NS as to type of crust | 1 |  | 1 | 1 | 1 | 1 | 1 | 1 |
| Pizza with extra meat and extra vegetables, regular crust | 1 |  | 1 | 1 | 1 | 1 | 1 | 1 |
| Pizza with extra meat and extra vegetables, thick crust | 1 |  | 1 | 1 | 1 | 1 | 1 | 1 |
| Pizza with extra meat and extra vegetables, thin crust | 0 |  | 1 | 0 | 0 | 0 | 1 | 1 |
| Pizza with extra meat, NS as to type of crust | 0 |  | 1 | 0 | 1 | 0 | 1 | 1 |
| Pizza with extra meat, regular crust | 0 |  | 1 | 0 | 1 | 0 | 1 | 1 |
| Pizza with extra meat, thick crust | 0 |  | 1 | 0 | 1 | 0 | 1 | 1 |
| Pizza with extra meat, thin crust | 0 |  | 0 | 0 | 0 | 0 | 1 | 1 |
| Pizza with meat and fruit, NS as to type of crust | 0 |  | 1 | 1 | 1 | 0 | 1 | 1 |
| Pizza with meat and fruit, regular crust | 0 |  | 1 | 1 | 1 | 0 | 1 | 1 |
| Pizza with meat and fruit, thick crust | 0 |  | 1 | 1 | 1 | 0 | 1 | 1 |
| Pizza with meat and fruit, thin crust | 0 |  | 1 | 0 | 0 | 0 | 1 | 1 |
| Pizza with meat and vegetables, NS as to type of crust | 0 |  | 1 | 0 | 1 | 0 | 1 | 1 |
| Pizza with meat and vegetables, prepared from frozen, thick crust | 0 |  | 1 | 1 | 1 | 0 | 1 | 1 |
| Pizza with meat and vegetables, prepared from frozen, thin crust | 0 |  | 1 | 0 | 1 | 1 | 1 | 1 |
| Pizza with meat and vegetables, regular crust | 0 |  | 1 | 0 | 1 | 0 | 1 | 1 |
| Pizza with meat and vegetables, thick crust | 0 |  | 1 | 1 | 1 | 0 | 1 | 1 |
| Pizza with meat and vegetables, thin crust | 0 |  | 1 | 0 | 0 | 0 | 1 | 1 |
| Pizza with meat other than pepperoni, from restaurant or fast food, NS as to type of crust | 0 |  | 1 | 1 | 1 | 0 | 1 | 1 |
| Pizza with meat other than pepperoni, from restaurant or fast food, regular crust | 0 |  | 1 | 1 | 1 | 0 | 1 | 1 |
| Pizza with meat other than pepperoni, from restaurant or fast food, thick crust | 0 |  | 1 | 0 | 1 | 0 | 1 | 1 |
| Pizza with meat other than pepperoni, from restaurant or fast food, thin crust | 0 |  | 0 | 0 | 0 | 0 | 1 | 1 |
| Pizza with meat, prepared from frozen, thick crust | 0 |  | 1 | 1 | 1 | 0 | 1 | 1 |
| Pizza with meat, prepared from frozen, thin crust | 0 |  | 1 | 0 | 1 | 1 | 1 | 1 |
| Pizza with pepperoni, from restaurant or fast food, NS as to type of crust | 0 |  | 1 | 1 | 1 | 0 | 1 | 1 |
| Pizza with pepperoni, from restaurant or fast food, regular crust | 0 |  | 1 | 1 | 1 | 0 | 1 | 1 |
| Pizza with pepperoni, from restaurant or fast food, thick crust | 0 |  | 0 | 1 | 1 | 0 | 1 | 1 |
| Pizza with pepperoni, from restaurant or fast food, thin crust | 0 |  | 0 | 0 | 0 | 0 | 1 | 1 |
| Pizza with pepperoni, from school lunch, thick crust | 0 |  | 1 | 0 | 1 | 0 | 1 | 1 |
| Pizza with pepperoni, from school lunch, thin crust | 1 |  | 1 | 1 | 1 | 1 | 1 | 1 |
| Pizza with pepperoni, stuffed crust | 0 |  | 0 | 0 | 0 | 0 | 1 | 1 |
| Pizza, cheese with vegetables, prepared from frozen, thick crust | 1 |  | 1 | 1 | 1 | 1 | 1 | 1 |
| Pizza, cheese, from restaurant or fast food, NS as to type of crust | 0 |  | 1 | 1 | 1 | 0 | 1 | 1 |
| Pizza, cheese, from restaurant or fast food, regular crust | 0 |  | 1 | 1 | 1 | 0 | 1 | 1 |
| Pizza, cheese, from restaurant or fast food, thick crust | 0 |  | 1 | 1 | 1 | 0 | 1 | 1 |
| Pizza, cheese, from restaurant or fast food, thin crust | 0 |  | 0 | 0 | 0 | 0 | 1 | 1 |
| Pizza, cheese, from school lunch, thick crust | 0 |  | 1 | 1 | 0 | 1 | 1 | 1 |
| Pizza, cheese, from school lunch, thin crust | 1 |  | 1 | 1 | 1 | 1 | 1 | 1 |
| Pizza, cheese, prepared from frozen, thick crust | 1 |  | 1 | 1 | 1 | 1 | 1 | 1 |
| Pizza, cheese, prepared from frozen, thin crust | 0 |  | 1 | 1 | 0 | 1 | 1 | 1 |
| Pizza, cheese, stuffed crust | 0 |  | 1 | 1 | 0 | 0 | 1 | 1 |
| Pizza, cheese, with fruit, NS as to type of crust | 1 |  | 1 | 1 | 1 | 1 | 1 | 1 |
| Pizza, cheese, with fruit, regular crust | 1 |  | 1 | 1 | 1 | 1 | 1 | 1 |
| Pizza, cheese, with fruit, thick crust | 1 |  | 1 | 1 | 1 | 1 | 1 | 1 |
| Pizza, cheese, with fruit, thin crust | 0 |  | 1 | 0 | 0 | 0 | 1 | 1 |
| Pizza, cheese, with vegetables, NS as to type of crust | 1 |  | 1 | 1 | 1 | 1 | 1 | 1 |
| Pizza, cheese, with vegetables, prepared from frozen, thin crust | 0 |  | 1 | 0 | 1 | 1 | 1 | 1 |
| Pizza, cheese, with vegetables, regular crust | 1 |  | 1 | 1 | 1 | 1 | 1 | 1 |
| Pizza, cheese, with vegetables, thick crust | 1 |  | 1 | 1 | 1 | 1 | 1 | 1 |
| Pizza, cheese, with vegetables, thin crust | 0 |  | 1 | 0 | 0 | 0 | 1 | 1 |
| Pizza, extra cheese, NS as to type of crust | 0 |  | 1 | 1 | 1 | 0 | 1 | 1 |
| Pizza, extra cheese, regular crust | 0 |  | 1 | 1 | 1 | 0 | 1 | 1 |
| **S1 Table (continued)** |  |  |  | | |  |  |  |
|  | **Overall** |  | **Specific nutrient targets** | | |  |  |  |
| **Pizza name** | **NNPS Outcome** |  | **Energy (kcal)** | **Total fat** | **Saturated fat** | **Sodium** | **Added sugars** | **Protein** |
| Pizza, extra cheese, thick crust | 0 |  | 1 | 1 | 1 | 0 | 1 | 1 |
| Pizza, extra cheese, thin crust | 0 |  | 0 | 0 | 0 | 0 | 1 | 1 |
| Pizza, no cheese, thick crust | 0 |  | 0 | 1 | 1 | 1 | 1 | 0 |
| Pizza, no cheese, thin crust | 0 |  | 1 | 0 | 1 | 0 | 1 | 0 |
| Pizza, with cheese and extra vegetables, NS as to type of crust | 1 |  | 1 | 1 | 1 | 1 | 1 | 1 |
| Pizza, with meat other than pepperoni, from school lunch, thick crust | 1 |  | 1 | 1 | 1 | 1 | 1 | 1 |
| Pizza, with meat other than pepperoni, from school lunch, thin crust | 1 |  | 1 | 1 | 1 | 1 | 1 | 1 |
| Pizza, with meat other than pepperoni, stuffed crust | 0 |  | 1 | 0 | 0 | 0 | 1 | 1 |
| White pizza, thick crust | 0 |  | 0 | 0 | 1 | 1 | 1 | 1 |
| White pizza, thin crust | 0 |  | 0 | 0 | 0 | 1 | 1 | 1 |
| Calzone, with cheese, meatless | 0 |  | 0 | 0 | 0 | 0 | 1 | 1 |
| Calzone, with meat and cheese | 0 |  | 0 | 0 | 1 | 1 | 1 | 1 |
