# Supplementary material for: Modeled Dietary Impact of Pizza Reformulations in US Children and Adolescents
Source: PLoS One. 2016 Oct 5;11(10):e0164197. doi: 10.1371/journal.pone.0164197 (PMC5051708; doi:10.1371/journal.pone.0164197)
Supplement: S2 Table — (DOCX) [file pone.0164197.s002.docx]

**Supporting Information - S2 Table**

| **Pizza Substitution Table** |  |
| --- | --- |
| **NNPS_Fail (Original food code)** | **NNPS_Pass (Food code used in the NNPS substitution scenario)** |
| Pizza with cheese and extra vegetables, thin crust | Pizza, cheese, prepared from frozen, thick crust |
| Pizza with extra meat and extra vegetables, thin crust | Pizza, cheese, prepared from frozen, thick crust |
| Pizza with extra meat, NS as to type of crust | Pizza, cheese, prepared from frozen, thick crust |
| Pizza with extra meat, regular crust | Pizza, cheese, prepared from frozen, thick crust |
| Pizza with extra meat, thick crust | Pizza, cheese, prepared from frozen, thick crust |
| Pizza with extra meat, thin crust | Pizza, cheese, prepared from frozen, thick crust |
| Pizza with meat and fruit, NS as to type of crust | Pizza, cheese, prepared from frozen, thick crust |
| Pizza with meat and fruit, regular crust | Pizza, cheese, prepared from frozen, thick crust |
| Pizza with meat and fruit, thick crust | Pizza, cheese, prepared from frozen, thick crust |
| Pizza with meat and fruit, thin crust | Pizza, cheese, prepared from frozen, thick crust |
| Pizza with meat and vegetables, NS as to type of crust | Pizza, cheese, prepared from frozen, thick crust |
| Pizza with meat and vegetables, prepared from frozen, thick crust | Pizza, cheese, prepared from frozen, thick crust |
| Pizza with meat and vegetables, prepared from frozen, thin crust | Pizza, cheese, prepared from frozen, thick crust |
| Pizza with meat and vegetables, regular crust | Pizza, cheese, prepared from frozen, thick crust |
| Pizza with meat and vegetables, thick crust | Pizza, cheese, prepared from frozen, thick crust |
| Pizza with meat and vegetables, thin crust | Pizza, cheese, prepared from frozen, thick crust |
| Pizza with meat other than pepperoni, from restaurant or fast food, NS as to type of crust | Pizza, cheese, prepared from frozen, thick crust |
| Pizza with meat other than pepperoni, from restaurant or fast food, regular crust | Pizza, cheese, prepared from frozen, thick crust |
| Pizza with meat other than pepperoni, from restaurant or fast food, thick crust | Pizza, cheese, prepared from frozen, thick crust |
| Pizza with meat other than pepperoni, from restaurant or fast food, thin crust | Pizza, cheese, prepared from frozen, thick crust |
| Pizza with meat, prepared from frozen, thick crust | Pizza, cheese, prepared from frozen, thick crust |
| Pizza with meat, prepared from frozen, thin crust | Pizza with pepperoni, from school lunch, thin crust |
| Pizza with pepperoni, from restaurant or fast food, NS as to type of crust | Pizza, cheese, prepared from frozen, thick crust |
| Pizza with pepperoni, from restaurant or fast food, regular crust | Pizza, cheese, prepared from frozen, thick crust |
| Pizza with pepperoni, from restaurant or fast food, thick crust | Pizza, cheese, prepared from frozen, thick crust |
| Pizza with pepperoni, from restaurant or fast food, thin crust | Pizza, cheese, prepared from frozen, thick crust |
| Pizza with pepperoni, from school lunch, thick crust | Pizza, cheese, prepared from frozen, thick crust |
| Pizza with pepperoni, stuffed crust | Pizza, cheese, prepared from frozen, thick crust |
| Pizza, cheese, from restaurant or fast food, NS as to type of crust | Pizza, cheese, prepared from frozen, thick crust |
| Pizza, cheese, from restaurant or fast food, regular crust | Pizza, cheese, prepared from frozen, thick crust |
| Pizza, cheese, from restaurant or fast food, thick crust | Pizza, cheese, prepared from frozen, thick crust |
| Pizza, cheese, from restaurant or fast food, thin crust | Pizza, cheese, prepared from frozen, thick crust |
| Pizza, cheese, from school lunch, thick crust | Pizza, with meat other than pepperoni, from school lunch, thin crust |
| Pizza, cheese, prepared from frozen, thin crust | Pizza, cheese, from school lunch, thin crust |
| Pizza, cheese, stuffed crust | Pizza, cheese, prepared from frozen, thick crust |
| Pizza, cheese, with fruit, thin crust | Pizza, cheese, prepared from frozen, thick crust |
| Pizza, cheese, with vegetables, prepared from frozen, thin crust | Pizza, with meat other than pepperoni, from school lunch, thin crust |
| Pizza, cheese, with vegetables, thin crust | Pizza, cheese, prepared from frozen, thick crust |
| Pizza, extra cheese, NS as to type of crust | Pizza, cheese, prepared from frozen, thick crust |
| Pizza, extra cheese, regular crust | Pizza, cheese, prepared from frozen, thick crust |
| Pizza, extra cheese, thick crust | Pizza, cheese, prepared from frozen, thick crust |
| Pizza, extra cheese, thin crust | Pizza, cheese, prepared from frozen, thick crust |
| Pizza, no cheese, thick crust | Pizza, with meat other than pepperoni, from school lunch, thick crust |
| Pizza, no cheese, thin crust | Pizza, cheese, prepared from frozen, thick crust |
| Pizza, with meat other than pepperoni, stuffed crust | Pizza, cheese, prepared from frozen, thick crust |
| White pizza, thick crust | Pizza, with meat other than pepperoni, from school lunch, thick crust |
| White pizza, thin crust | Pizza, with meat other than pepperoni, from school lunch, thick crust |
| Calzone, with cheese, meatless | Pizza, with meat other than pepperoni, from school lunch, thick crust |
| Calzone, with meat and cheese | Pizza, cheese, from school lunch, thin crust |
