# Supplementary material for: Modeled Dietary Impact of Pizza Reformulations in US Children and Adolescents
Source: PLoS One. 2016 Oct 5;11(10):e0164197. doi: 10.1371/journal.pone.0164197 (PMC5051708; doi:10.1371/journal.pone.0164197)
Supplement: S3 Table — (DOCX) [file pone.0164197.s003.docx]

**Supporting Information - S3 Table**

| **Prevalence of pizza consumption in NHANES 2011-12 children and adolescents having completed the first 24-hr recall (Day 1), by age group, gender, education level, and ethnicity** | | | | | |
| --- | --- | --- | --- | --- | --- |
|  |  |  | 4-11 years | | 12-19 years |
| Total | Total n |  | 1503 |  | 1152 |
|  | Prevalence (%)^a^ |  | 20.6 |  | 21.1 |
|  |  |  |  |  |  |
| Females | Total n |  | 731 |  | 567 |
|  | Prevalence (%)^a^ |  | 21.5 |  | 20.2 |
| Males | Total n |  | 772 |  | 585 |
|  | Prevalence (%)^a^ |  | 22.1 |  | 20.8 |
|  |  |  |  |  |  |
| Household education, up to high school^b^ | Total n |  | 1118 |  | 882 |
|  | Prevalence (%)^a^ |  | 18.1 |  | 21.7 |
| Household education, College and above^b^ | Total n |  | 347 |  | 232 |
|  | Prevalence (%)^a^ |  | 27 |  | 18.2 |
| Ethnicity^c^ |  |  |  |  |  |
| White | Total n |  | 346 |  | 259 |
|  | Prevalence (%)^a^ |  | 21.8 |  | 22 |
| Mexican | Total n |  | 306 |  | 198 |
|  | Prevalence (%)^a^ |  | 18.1 |  | 24.5 |
| Black | Total n |  | 432 |  | 352 |
|  | Prevalence (%)^a^ |  | 18.5 |  | 19.2 |
| Asian | Total n |  | 159 |  | 158 |
|  | Prevalence (%)^a^ |  | 20 |  | 17.1 |
| ^a^ Pizza consumption was defined as having declared consuming a pizza food code at least once during Day 1. % were adjusted based on sampling design, using the ‘survey’ package of R | | | | | |
| ^b^ Self-reported education level of the head of the household | |  |  |  |  |
| ^c^ Ethnicity was self-reported |  |  |  |  |  |
