# Supplementary material for: Modeled Dietary Impact of Pizza Reformulations in US Children and Adolescents
Source: PLoS One. 2016 Oct 5;11(10):e0164197. doi: 10.1371/journal.pone.0164197 (PMC5051708; doi:10.1371/journal.pone.0164197)
Supplement: S4 Table — (DOCX) [file pone.0164197.s004.docx]

**Supporting Information - S4 Table**

| **Mean nutrient content per 100 g of pizza food codes in the FNDDS 2011-12 database, by NNPS standards ^a^** | | | | | | | | | | |
| --- | --- | --- | --- | --- | --- | --- | --- | --- | --- | --- |
|  |  | |  |  | |  |  | |  |  |
|  | **All pizzas  (n=69)** | |  | **Pizzas not meeting NNPS standards (n=49)** | |  | **Pizzas meeting NNPS  standards (n=20)** | |  |  |
| **Nutrient** | **Mean** | **SE** |  | **Mean** | **SE** |  | **Mean** | **SE** |  | **p_Fail-Pass_** |
| Energy (kcal) | 268 | 4.03 |  | 280 | 4.66 |  | 241 | 3.31 |  | <.001 |
| Total fat (g) | 11.6 | 0.352 |  | 12.7 | 0.403 |  | 9.09 | 0.2 |  | <.001 |
| Monounsaturated fats (g) | 3.62 | 0.172 |  | 4.05 | 0.208 |  | 2.56 | 0.114 |  | <.001 |
| Polyunsaturated fats (g) | 2.14 | 0.0697 |  | 2.26 | 0.0844 |  | 1.86 | 0.0994 |  | 0.00336 |
| Saturated fats (g) | 4.78 | 0.158 |  | 5.21 | 0.188 |  | 3.72 | 0.0874 |  | <.001 |
| Carbohydrates (g) | 29.2 | 0.36 |  | 29.1 | 0.469 |  | 29.3 | 0.491 |  | 0.817 |
| Total sugars (g) | 3.75 | 0.144 |  | 3.55 | 0.157 |  | 4.24 | 0.29 |  | 0.0462 |
| Added sugars (g) | 1.06 | 0.137 |  | 0.97 | 0.139 |  | 1.29 | 0.33 |  | 0.381 |
| Protein (g) | 11.7 | 0.221 |  | 12.1 | 0.271 |  | 10.7 | 0.274 |  | <.001 |
| Fibers (g) | 2.12 | 0.0456 |  | 2.07 | 0.0483 |  | 2.26 | 0.0993 |  | 0.0919 |
| Calcium (mg) | 181 | 6.8 |  | 187 | 9.1 |  | 164 | 6.13 |  | 0.0395 |
| Iron (mg) | 2.08 | 0.038 |  | 2.06 | 0.0499 |  | 2.13 | 0.0477 |  | 0.302 |
| Potassium (mg) | 186 | 3.65 |  | 186 | 4.23 |  | 186 | 7.33 |  | 0.965 |
| Magnesium (mg) | 23.4 | 0.502 |  | 22.9 | 0.335 |  | 24.8 | 1.51 |  | 0.235 |
| Sodium (mg) | 586 | 10.7 |  | 614 | 13 |  | 517 | 5.69 |  | <.001 |
| Niacin (mg) | 3.12 | 0.0864 |  | 3.16 | 0.107 |  | 3 | 0.14 |  | 0.36 |
| Phosphorus (mg) | 211 | 4.87 |  | 216 | 6.4 |  | 198 | 5.24 |  | 0.0316 |
| Zinc (mg) | 1.38 | 0.0292 |  | 1.44 | 0.0358 |  | 1.24 | 0.032 |  | <.001 |
| Vitamin A (mg RAE) | 64.7 | 2.51 |  | 67.6 | 3.36 |  | 57.5 | 1.96 |  | 0.0114 |
| Thiamin (mg) | 0.304 | 0.00956 |  | 0.307 | 0.0125 |  | 0.295 | 0.0126 |  | 0.489 |
| Riboflavin (mg) | 0.21 | 0.007 |  | 0.218 | 0.00914 |  | 0.189 | 0.00747 |  | 0.0163 |
| Vitamin B6 (mg) | 0.0958 | 0.00326 |  | 0.0981 | 0.00404 |  | 0.09 | 0.00528 |  | 0.228 |
| Folate (g) | 76.7 | 2.18 |  | 78.5 | 2.46 |  | 72.2 | 4.41 |  | 0.217 |
| Vitamin B12 (mcg) | 0.467 | 0.0199 |  | 0.505 | 0.0235 |  | 0.372 | 0.0284 |  | <.001 |
| Vitamin C (mg) | 2.44 | 0.228 |  | 2.24 | 0.28 |  | 2.92 | 0.376 |  | 0.155 |
| Vitamin D (mcg) | 0.0435 | 0.00812 |  | 0.0469 | 0.0101 |  | 0.035 | 0.0131 |  | 0.476 |
| Vitamin E (mg) | 0.873 | 0.0247 |  | 0.923 | 0.0315 |  | 0.753 | 0.0183 |  | <.001 |

^a^ NNPS: Nestlé Nutritional Profiling System.

RAE: Retinol equivalent
