# Supplementary material for: Modeled Dietary Impact of Pizza Reformulations in US Children and Adolescents
Source: PLoS One. 2016 Oct 5;11(10):e0164197. doi: 10.1371/journal.pone.0164197 (PMC5051708; doi:10.1371/journal.pone.0164197)
Supplement: S5 Table — (DOCX) [file pone.0164197.s005.docx]

**Supporting Information - S5 Table**

| **Mean nutrient content per 100 kcal or as % energy of pizza food codes in the FNDDS 2011-12 database, by NNPS standards ^a^** | | | | | | | | | | |
| --- | --- | --- | --- | --- | --- | --- | --- | --- | --- | --- |
|  |  | |  |  | |  |  | |  |  |
|  | **All pizzas  (n=69)** | |  | **Pizzas not meeting NNPS standards (n=49)** | |  | **Pizzas meeting NNPS  standards (n=20)** | |  |  |
| **Nutrient (/100kcal or as %energy)** | **Mean** | **SE** |  | **Mean** | **SE** |  | **Mean** | **SE** |  | **p_Fail-Pass_** |
| Total fat (% energy) | 0.386 | 0.0061 |  | 0.405 | 0.00664 |  | 0.339 | 0.00514 |  | <.001 |
| Saturated fats (% energy) | 0.159 | 0.00326 |  | 0.167 | 0.00384 |  | 0.139 | 0.00346 |  | <.001 |
| Total sugars (% energy) | 0.0572 | 0.00235 |  | 0.0521 | 0.00248 |  | 0.0698 | 0.00428 |  | 0.001 |
| Added sugars (% energy) | 0.0157 | 0.00193 |  | 0.0136 | 0.00179 |  | 0.0207 | 0.00492 |  | 0.193 |
| Protein (% energy) | 0.175 | 0.00241 |  | 0.174 | 0.00303 |  | 0.178 | 0.00376 |  | 0.425 |
| Fibers (g) | 0.809 | 0.0236 |  | 0.754 | 0.0228 |  | 0.946 | 0.0479 |  | 0.001 |
| Calcium (mg) | 67.3 | 2.16 |  | 66.9 | 2.84 |  | 68.4 | 2.71 |  | 0.699 |
| Iron (mg) | 0.787 | 0.0179 |  | 0.746 | 0.0206 |  | 0.889 | 0.024 |  | 0.000 |
| Folate (g) | 28.9 | 0.87 |  | 28.3 | 0.923 |  | 30.2 | 1.98 |  | 0.387 |
| Potassium (mg) | 70.5 | 1.73 |  | 67.6 | 1.84 |  | 77.5 | 3.51 |  | 0.018 |
| Magnesium (mg) | 8.87 | 0.249 |  | 8.28 | 0.17 |  | 10.3 | 0.655 |  | 0.007 |
| Sodium (mg) | 220 | 3.63 |  | 222 | 4.96 |  | 215 | 3.06 |  | 0.268 |
| Niacin (mg) | 1.18 | 0.0352 |  | 1.15 | 0.042 |  | 1.25 | 0.0633 |  | 0.169 |
| Phosphorus (mg) | 79 | 1.7 |  | 77.6 | 2.17 |  | 82.3 | 2.37 |  | 0.156 |
| Zinc (mg) | 0.517 | 0.00949 |  | 0.518 | 0.0119 |  | 0.515 | 0.0154 |  | 0.897 |
| Vitamin A (mg RAE) | 24.1 | 0.742 |  | 24.1 | 0.983 |  | 24 | 0.904 |  | 0.962 |
| Thiamin (mg) | 0.114 | 0.00371 |  | 0.111 | 0.00464 |  | 0.123 | 0.00562 |  | 0.101 |
| Riboflavin (mg) | 0.078 | 0.00203 |  | 0.0779 | 0.0027 |  | 0.0782 | 0.00238 |  | 0.925 |
| Vitamin B6 (mg) | 0.0362 | 0.00129 |  | 0.0357 | 0.00158 |  | 0.0375 | 0.00228 |  | 0.520 |
| Vitamin B12 (mcg) | 0.175 | 0.00723 |  | 0.184 | 0.00886 |  | 0.154 | 0.0113 |  | 0.049 |
| Vitamin C (mg) | 0.951 | 0.0928 |  | 0.834 | 0.109 |  | 1.24 | 0.164 |  | 0.047 |
| Vitamin D (mcg) | 0.0152 | 0.00265 |  | 0.0157 | 0.00313 |  | 0.0139 | 0.00506 |  | 0.763 |
| Vitamin E (mg) | 0.326 | 0.00777 |  | 0.33 | 0.0101 |  | 0.314 | 0.0104 |  | 0.279 |

^a^ NNPS: Nestlé Nutritional Profiling System.

RAE: Retinol equivalent
