# Supplementary material for: Modeled Dietary Impact of Pizza Reformulations in US Children and Adolescents
Source: PLoS One. 2016 Oct 5;11(10):e0164197. doi: 10.1371/journal.pone.0164197 (PMC5051708; doi:10.1371/journal.pone.0164197)
Supplement: S6 Table — (DOCX) [file pone.0164197.s006.docx]

**Supporting Information - S6 Table**

| **Mean nutritional intakes in NHANES 2011-12 children and adolescents aged 4 to 19 years consuming pizza^a^ (Day 1), at baseline and in the reformulation and substitution scenarios** | | | | | | | | | | |
| --- | --- | --- | --- | --- | --- | --- | --- | --- | --- | --- |
|  |  |  |  |  |  |  |  |  |  |  |
| **Nutrient** | **Gender and age group** | **Baseline** |  |  | **Reformulation scenario^b^** | | | **Substitution scenario^c^** | | |
|  |  | **Mean** | **SE** |  | **Mean** | **SE** |  | **Mean** | **SE** |  |
| Energy (kcal) | Males, 4-11y (n=182) | 2070 | 76.6 |  | 2059 | 77.0 | * | 2027 | 74.4 | * |
|  | Males, 12-19y (n=137) | 2685 | 96.0 |  | 2669 | 93.9 | * | 2627 | 91.1 | * |
|  | Females, 4-11y (n=146) | 1944 | 90.2 |  | 1917 | 78.9 | * | 1893 | 73.7 | * |
|  | Females, 12-19y (n=114) | 1936 | 169 |  | 1931 | 170.0 | * | 1909 | 168 | * |
|  |  |  |  |  |  |  |  |  |  |  |
| Total fat (g) | Males, 4-11y | 73.7 | 3.21 |  | 72.7 | 3.15 | * | 67.2 | 2.83 | * |
|  | Males, 12-19y | 100 | 4.88 |  | 98.0 | 4.66 | * | 90.3 | 4.10 | * |
|  | Females, 4-11y | 72.8 | 4.59 |  | 71.1 | 3.97 | * | 66.3 | 2.87 | * |
|  | Females, 12-19y | 69.9 | 9.16 |  | 69.2 | 9.14 | * | 64.8 | 8.77 | * |
|  |  |  |  |  |  |  |  |  |  |  |
| Saturated fat (g) | Males, 4-11y | 27.1 | 1.05 |  | 26.9 | 1.06 | * | 24.5 | 0.99 | * |
|  | Males, 12-19y | 35.4 | 1.83 |  | 35.1 | 1.78 | * | 31.9 | 1.57 | * |
|  | Females, 4-11y | 26.4 | 1.92 |  | 25.9 | 1.66 | * | 23.6 | 1.09 | * |
|  | Females, 12-19y | 25.2 | 3.67 |  | 25.1 | 3.69 | * | 23.4 | 3.57 | * |
|  |  |  |  |  |  |  |  |  |  |  |
| Sodium (mg) | Males, 4-11y | 3275 | 134 |  | 3135 | 127 | * | 3127 | 128 | * |
|  | Males, 12-19y | 4642 | 239 |  | 4420 | 223 | * | 4406 | 221 | * |
|  | Females, 4-11y | 3035 | 147 |  | 2923 | 135 | * | 2914 | 135 | * |
|  | Females, 12-19y | 3140 | 322 |  | 3046 | 314 | * | 3037 | 313 | * |
|  |  |  |  |  |  |  |  |  |  |  |
| ^a^ Pizza consumption was defined as having declared consuming a pizza food code at least once during Day 1. | | | | | | | |  |  |  |
| ^b^ In the Reformulation scenario, if the nutrient content of a pizza was not consistent with NNPS target, it was set to the NNPS target for this nutrient | | | | | | | | | | |
| c In the substitution scenario, all pizzas not consistent with NNPS standards were replaced by the closest pizza consistent with NNPS, based on a Euclidean distance calculated using all NNPS nutritional factors. | | | | | | | | | | |
| NNPS, Nestlé Nutritional Profiling System. The NNPS defines category-specific nutrient targets per portion size. All targets need to be met to be consistent with the NNPS standards. | | | | | | | | | | |
| * Due to the modeling, all differences in nutrient intakes between baseline and the reformulation scenario were highly significant (p <.001). | | | | | | | | | | |
