# Supplementary material for: Modeled Dietary Impact of Pizza Reformulations in US Children and Adolescents
Source: PLoS One. 2016 Oct 5;11(10):e0164197. doi: 10.1371/journal.pone.0164197 (PMC5051708; doi:10.1371/journal.pone.0164197)
Supplement: S7 Table — (DOCX) [file pone.0164197.s007.docx]

**Supporting Information - S7 Table**

| **Mean nutritional intakes expressed in percent of energy in NHANES 2011-12 children and adolescents aged 4 to 19 years (Day 1), at baseline and in the reformulation and substitution scenarios** | | | | | | | | | |
| --- | --- | --- | --- | --- | --- | --- | --- | --- | --- |
|  | Total sample (n=2655) | | | | | | | |  |
|  | Baseline | |  | Reformulation scenario ^a^ | | | Substitution scenario ^b^ | |  |
| Nutrient | **Mean** | **SE** |  | **Mean** | **SE** |  | **Mean** | **SE** |  |
| Energy (kcal) | 2049 | 26.3 |  | 2046 | 26.3 | * | 2040 | 26.0 | * |
| Total fat (g) | 76 | 1.58 |  | 75.6 | 1.59 | * | 74.6 | 1.53 | * |
| Total fat (% energy) | 32.7 | 0.28 |  | 32.6 | 0.28 | * | 32.2 | 0.28 | * |
| Saturated fat (g) | 26.2 | 0.5 |  | 26.1 | 0.50 | * | 25.6 | 0.49 | * |
| Saturated fat (% energy) | 11.3 | 0.11 |  | 11.2 | 0.11 | * | 11.1 | 0.12 | * |
| Sodium (mg) | 3274 | 56 |  | 3244 | 53.9 | * | 3242 | 53.8 | * |
| Protein (g) | 72.8 | 1.11 |  | 72.9 | 1.11 | * | 72.9 | 1.14 | * |
| Protein (% energy) | 14.3 | 0.16 |  | 14.3 | 0.16 | * | 14.4 | 0.17 | * |
| Total sugars (g) | 131 | 2.18 |  | 131 | 2.18 | * | 132 | 2.15 | * |
| Total sugars (% energy) | 26.1 | 0.46 |  | 26.2 | 0.47 | * | 26.4 | 0.46 | * |
| Added sugars (g) | 84.3 | 1.95 |  | 84.2 | 1.95 | * | 84.3 | 1.93 | * |
| Added sugars (% energy) | 16.5 | 0.29 |  | 16.5 | 0.29 | * | 16.6 | 0.29 | * |
|  |  |  |  |  |  |  |  |  |  |
| ^a^ In the Reformulation scenario, if the nutrient content of a pizza was not consistent with NNPS target, it was set to the NNPS target for this nutrient | | | | | | | | | |
| ^b^ In the substitution scenario, all pizzas not consistent with NNPS standards were replaced by the closest pizza consistent with NNPS, based on a Euclidean distance calculated using all NNPS nutritional factors. | | | | | | | | | |
| NNPS, Nestlé Nutritional Profiling System. The NNPS defines category-specific nutrient targets per portion size. All targets need to be met to be consistent with the NNPS standards. | | | | | | | | | |
| * Due to the modeling, all differences in nutrient intakes between baseline and the reformulation scenario were highly significant (p <.001). | | | | | | | | | |
